# Same Test, Better Scores: Boosting the Reliability of Short Online Intelligence Recruitment Tests with Nested Logit Item Response Theory Models

**DOI:** 10.3390/jintelligence7030017

**Published:** 2019-07-10

**Authors:** Martin Storme, Nils Myszkowski, Simon Baron, David Bernard

**Affiliations:** 1 IESEG School of Management, 59800 Lille, France; 2 LEM-CNRS 9221, 59800 Lille, France; 3 Department of Psychology, Pace University, New York, NY 10038, USA; 4 Assess First, 75000 Paris, France

**Keywords:** E-assessment, general mental ability, nested logit models, item-response theory, ability-based guessing

## Abstract

Assessing job applicants’ general mental ability online poses psychometric challenges due to the necessity of having brief but accurate tests. Recent research (Myszkowski & Storme, 2018) suggests that recovering distractor information through Nested Logit Models (NLM; Suh & Bolt, 2010) increases the reliability of ability estimates in reasoning matrix-type tests. In the present research, we extended this result to a different context (online intelligence testing for recruitment) and in a larger sample (N=2949 job applicants). We found that the NLMs outperformed the Nominal Response Model (Bock, 1970) and provided significant reliability gains compared with their binary logistic counterparts. In line with previous research, the gain in reliability was especially obtained at low ability levels. Implications and practical recommendations are discussed.

## 1. Introduction

With the development of the Internet, the assessment of job applicants is increasingly performed online, which facilitates large scale testing while reducing costs [1,2]. This recent trend has led to the creation of a new research field in psychometrics, referred to as *e-assessment* [2]. Considering the relevance of General Mental Ability (GMA) in predicting job performance [3], many e-assessment platforms have included tasks that aim at capturing it—such as logical series or logical reasoning matrices—in their online test batteries.

The assessment phase in e-recruiting poses very specific psychometric challenges. On the one hand, the assessment should ideally lead to a short-list of the best applicants [1,2]. The accuracy of the assessment is therefore a key issue in e-recruiting just like in it is in recruiting in general. On the other hand, the assessment phase cannot require from applicants that they take part in assessment processes that are too time consuming and too cognitively demanding. It is indeed not acceptable to extensively test people who have a relatively low chance of getting an interview. Perceived unfairness of the recruitment process has been shown to have a negative impact on the image of the recruiting company, which can lead to negative word of mouth and/or intentions not to complete the recruitment process [4,5]. The challenge that is inherent to e-assessment in a recruitment context is essentially the challenge of short psychometric measures, which is to extract as much information as possible from short instruments.

Extracting reliable information from short tests remains a real challenge from a measurement perspective [6]. Hopefully, psychometricians have allies in this challenging endeavour, such as Item Response Theory (IRT) modeling, which often allows them to extract more information from short psychometric tools than Classical Test Theory (CTT). Originally suggested for multiple-choice items by Bock [7], one way that researchers can take advantage of the IRT framework in logical series or matrices tests consists in extracting information from which incorrect responses were selected. This approach is based on the premise that when a test taker selects a wrong response option out of a set of wrong response options, the choice of the wrong response option can carry information about the ability of the test taker. Further, recent developments [8] applied to progressive matrices have suggested recovering additional information from distractor responses through Nested Logit Models (NLM) [9], and have indicated that such models may be more appropriate than Bock’s [7] Nominal Response Model in logical reasoning tests, but also than traditional binary IRT models [8]. In this research, recovering information from the choice of distractors has provided significant gains in reliability in comparison with not recovering such information and using traditional binary logistic models.

Currently, no study has investigated whether applying this approach in the field of recruitment would lead to gains in reliability. Yet, taking an online GMA test as part of a recruitment process is in several ways different from taking a GMA test for an experiment in the lab. There is reasonable evidence to suspect that such differences could affect the way distractors are processed by test takers, which could possibly jeopardize the very idea of recovering psychometric information from distractors. In the present article, our main aim is to extend and conceptually replicate previous research on students and in laboratory conditions [8] to online personnel pre-selection contexts, by testing whether the modeling strategies previously suggested are able, even in this context, to provide tangible gains in reliability. The effort of conducting conceptual replications in the field is crucial in psychology to rule out the possibility that a laboratory finding is too weak to be relevant in contexts that are less tightly controlled [10].

### 1.1. Binary Item Response Theory Models

Item Response Theory (IRT) has traditionally helped psychometricians improve the reliability of the ability estimates obtained with short intelligence measures [8,11]. IRT provides a framework that has indeed been shown to improve the reliability of measurement compared to the Classical Test Theory (CTT) approach [12]. While CTT assumes that all items are linked to the latent trait in a similar fashion, IRT assumes that each item is linked to the the latent trait in a unique manner [13]. The aim of IRT is to model the probability of a response to an item as a function of the latent trait or ability of the test taker, traditionally with a non-linear function of the latent trait that is unique for each item. In the case of binary responses, the non-linear function is, frequently, the logistic function. Because of the flexibility of its parametrization in comparison with CTT, IRT allows for the accounting of a variety of testing phenomena and extracting information that is relevant in the context of GMA assessment [8].

GMA tests, such as progressive matrices or logical series, usually contain one correct answer option and several incorrect answer options—which are often referred to as distractors. Although the response dataset is thus polytomous, it is typical to recode the dataset by collapsing the distractor responses together, which yields a dichotomous success/failure variable format. The binary IRT approach generally consists in modeling these dichotomous responses using a logistic function of the latent ability and a set of item parameters representing various item characteristics (difficulty, discrimination, etc.).

The simplest IRT models, including only one parameter and referred to as One-Parameter Logistic (1PL) models, characterize items by their level of difficulty only. The difficulty parameter corresponds to the level of the latent trait for which the slope of the function linking the ability and the probability to select the correct response option reaches its maximum—in other words, the ability level where the discrimination of the item is at its maximum. The model is often extended with another parameter—discrimination—leading to Two-Parameter Logistic (2PL) models. Such models not only take into account the difficulty of an item, but also its ability to discriminate between ability levels. The discrimination parameter corresponds to the strength of relationship between the ability and the probability to select the correct response option. Three-Parameter Logistic (3PL) models add to previous models a variable lower asymptote in the relation between the ability and the probability to select the correct response option. In the context of IRT, the lower asymptote corresponds to the probability to select the correct answer to a given item by guessing it. Therefore, 3PL models allow to characterize items regarding the extent to which they are susceptible to correct guessing. A fourth parameter is included in 4-Parameter Logistic (4PL) models, which corresponds to a variable upper asymptote in the relation between the ability and the probability to select the correct response option. In the context of IRT, the upper asymptote corresponds to the probability of responding incorrectly to an item in spite of having a level of ability that should normally lead to providing the right answer. This parameter allows the modelling of the phenomenon of inattention or slipping. Although 4PL models are used less frequently than 1, 2, and 3PL models, they have been shown to correct adequately for careless mistakes and to improve measurement efficiency [14,15].

Although binary IRT is able to model phenomena that appear in matrix-type reasoning tasks, even models that include guessing fail to account for the possibility that choosing a distractor over another one could be related to the respondent’s ability—a phenomenon often described as as ability-based guessing [16]. Indeed, the lower asymptote parameter of the 3PL and 4PL models account for the probability of correctly guessing, but what distractor is chosen when an examinee uses a guessing strategy is not considered—all distractor responses are still collapsed together as incorrect. Yet, if one considers that the guessing process is related to the ability, then the outcome of this process—the distractor chosen—can contain information about the ability that binary models fail to recover.

### 1.2. Recovering Distractor Information

In matrix-type or logical series type tests, distractors are usually designed in a way that they are only partially in line with the set of rules that structures the logical series. For example, if three rules are structuring the progression of a logical series, the correct response option will respect all three of them, but frequently a distractor could respect only two, while another may respect one or even none of them. In this example, a distractor that respects two out of three rules could be considered as a better response option than a distractor that would only respect one out of three rules, although both are ultimately incorrect response options. As a consequence, the wrong response options that are selected by test takers are usually not equivalent in (in)correctness, and thus could carry information about their ability [17].

#### 1.2.1. The Nominal Response Model

A traditional approach to recovering information from distractors is to fit the nominal data with Bock’s [7] Nominal Response Model (NRM). This model is essentially a multinomial adaptation of the 2PL model, where the probability $P_{iv}$ that an examinee *j* chooses a category *v*—which could be the correct response or a distractor—among the $m_{i}$ possible responses for item *i* is modeled as a function of the examinee’s ability $\theta_{j}$, an intercept item-category parameter $\zeta_{iv}$ and a slope item-category parameter $\lambda_{iv}$, as well as the item-category parameters of all other categories, such as:

(1) $$P_{iv}\left(\theta_{j}\right)=\frac{e^{\zeta_{iv}+\lambda_{iv}\theta_{j}}}{\sum_{k=1}^{m_{i}}e^{\zeta_{ik}+\lambda_{ik}\theta_{j}}}$$

A way to interpret this model is to essentially consider each category as having a propensity $e^{\zeta_{iv}+\lambda_{iv}\theta_{j}}$ and the probability of selecting a category depends on the category’s propensity over the total of all category propensities. When applied to multiple choice items, a consequence of this is that the Nominal Response Model’s formulation is mathematically consistent with a response process where all response categories compete with one another and where, depending on the examinee’s ability, one category would dominate in propensity the others, and result in the examinee responding (more probably) in favor of that category [9]. But, as we later discuss, this representation of the response process may not be in line with all multiple-choice tests, especially in the case of logical reasoning matrices or logical series.

#### 1.2.2. Nested Logit Models

In certain multiple-choice tests, in order to respond, the examinee is supposed to consider a stimulus (for example, in matrix-type tests, the incomplete matrix), from which a rule should be extracted and used to find the completing element. In such cases, it can be questioned whether examinees put into competition the different response options right away—a process that would ideally be modeled by the NRM. Instead, it could be that they first focus on understanding the stimulus (the matrix, or the beginning of the series) to find the correct response (regardless of what the response options are). From that process, two situations may arise—either they have understood the rule correctly and found the correct response—in that case, the distractors are not really considered as viable options and the correct response is selected—or they have not—and in that case the response options are put in competition in the guessing strategy.

Such a sequential process was described by Suh and Bolt [9] as a motivation to develop a new class of item-response models for multiple-choice questions where this double process could be considered: Nested Logit Models (NLM). NLMs have been designed to model situations in which the response choice possesses a nested structure, that is when the final choice of a response option is made through a sequential process.

NLMs attempt to approximate the response probabilities that occur from this sequential process and the two models that best describe each step into a single model. NLMs have two levels that separate the response options in two nests. At a higher level (level 1), the model distinguishes the choice of the correct response option versus the choice of any incorrect response option, which can be achieved with a binary logistic IRT model (e.g. the 2PL, 3PL or 4PL model). At a lower level (level 2), the model distinguishes the probability of selecting one particular distractor (as opposed to another one) as the product of the probability of selecting any distractor (which is the complement of the probability earlier modeled with the level 1 part) and a probability modeled using the propensities of each distractor—which is similar to a Nominal Response Model of the distractors.

To summarize, using the 4-Parameter NLM (4PNL) as an example for at level 1, the probability $P(x_{ij}=u|\theta_{j})$ that the *j*th person selects the correct response (category *u*) to the *i*th item, depends on their ability $\theta_{j}$ and item parameters $\alpha_{i}$ (discrimination/slope), $\beta_{i}$ (difficulty/intercept), $\gamma_{i}$ (lower asymptote) and $\delta_{i}$ (upper asymptote), such as:

(2) $$P\left(x_{ij}=u|\theta_{j}\right)=\gamma_{i}+\frac{\delta_{i}-\gamma_{i}}{1+e^{-\left(\beta_{i}+\alpha_{i}\theta_{j}\right)}}$$

Similar to binary logistic models, the 3-Parameter Nested Logit (3PNL) model is a constrained 4PNL where $\delta_{i}$ is fixed, generally (and throughout in this paper) to 1, such as:

(3) $$P\left(x_{ij}=u|\theta_{j}\right)=\gamma_{i}+\frac{1-\gamma_{i}}{1+e^{-\left(\beta_{i}+\alpha_{i}\theta_{j}\right)}}$$

Further, the 2-Parameter Nested Logit (2PNL) is a constrained 3PNL where $\gamma_{i}$ is fixed, generally (and throughout in this paper) to 0, such as:

(4) $$P\left(x_{ij}=u|\theta_{j}\right)=\frac{1}{1+e^{-\left(\beta_{i}+\alpha_{i}\theta_{j}\right)}}$$

At level 2, which models the distractor responses, the probability $P(x_{ij}=v|\theta_{j})$ that the examinee selects the distractor category *v* among the $m_{i}$ possible distractor responses is modeled as the product of the probability of responding incorrectly $1-P(x_{ij}=u|\theta_{j})$ and the probability that the examinee selects the distractor conditional upon an incorrect response. The latter is in fact similar to a Nominal Response model, where distractor responses have propensities that are a function of the ability $\theta_{j}$, intercept $\zeta_{iv}$ and slope $\lambda_{iv}$. The resulting distractor model for the probability $P\left(U_{ij}=0,D_{ijv}|\theta_{j}\right)$ that person *j* selects distractor *v* for item *i* is thus given by:

(5) $$P\left(x_{ij}=v|\theta_{j}\right)=\left[1-P(x_{ij}=u|\theta_{j})\right]\left[\frac{e^{\zeta_{iv}+\lambda_{iv}\theta_{j}}}{\sum_{k=1}^{m_{i}}e^{\xi_{ik}+\lambda_{ik}\theta_{j}}}\right]$$

Using the level 1 4PL model in Equation (2), the distractors-model results in the 4PNL model to:

(6) $$P\left(x_{ij}=v|\theta_{j}\right)=\left[1-\left(\gamma_{i}+\frac{\delta_{i}-\gamma_{i}}{1+e^{-\left(\beta_{i}+\alpha_{i}\theta_{j}\right)}}\right)\right]\left[\frac{e^{\zeta_{iv}+\lambda_{iv}\theta_{j}}}{\sum_{k=1}^{m_{i}}e^{\xi_{ik}+\lambda_{ik}\theta_{j}}}\right]$$

Using the level 1 3PL model in Equation (3), the distractors-model results in the 3PNL model to:

(7) $$P\left(x_{ij}=v|\theta_{j}\right)=\left[1-\left(\gamma_{i}+\frac{1-\gamma_{i}}{1+e^{-\left(\beta_{i}+\alpha_{i}\theta_{j}\right)}}\right)\right]\left[\frac{e^{\zeta_{iv}+\lambda_{iv}\theta_{j}}}{\sum_{k=1}^{m_{i}}e^{\xi_{ik}+\lambda_{ik}\theta_{j}}}\right]$$

Using the level 1 2PL model in Equation (4), the distractors-model results in the 2PNL model to:

(8) $$P\left(x_{ij}=v|\theta_{j}\right)=\left[1-\frac{1}{1+e^{-\left(\beta_{i}+\alpha_{i}\theta_{j}\right)}}\right]\left[\frac{e^{\zeta_{iv}+\lambda_{iv}\theta_{j}}}{\sum_{k=1}^{m_{i}}e^{\xi_{ik}+\lambda_{ik}\theta_{j}}}\right]$$

An important distinction to note between the models of this class and the Nominal Response Model is that, in the NLM, the probability of a correct response is not directly affected by the propensities towards the different distractors, but the probability to select the distractors is conditional upon the probability of a correct (or rather, incorrect) response. In contrast, in the Nominal Response Model, the propensities towards all response categories—correct response and distractors alike—all affect one another.

**Figure 1:**
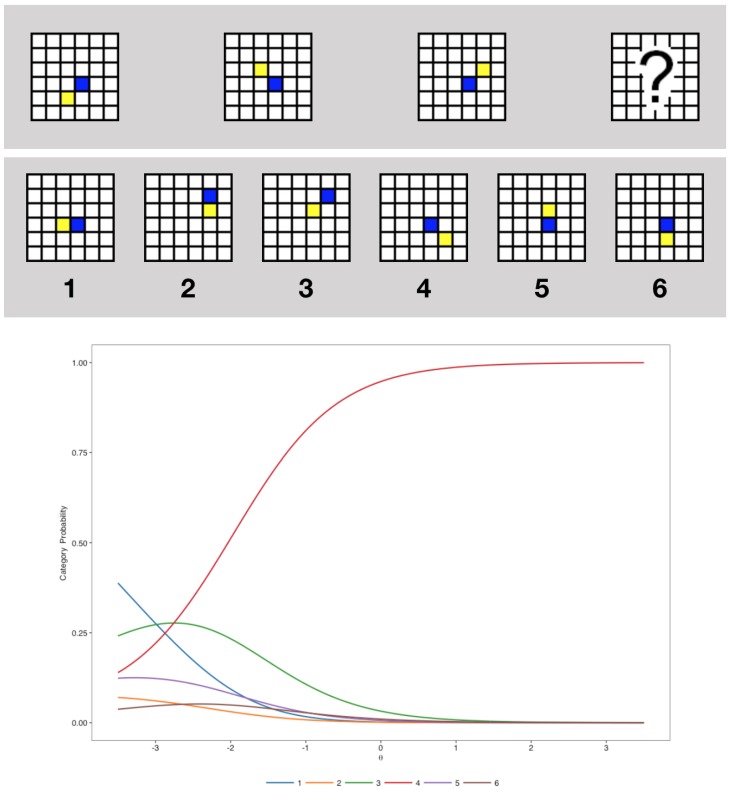
An example item of the GF20 (**top**) and the associated category characteristic curves as estimated by the 3-Parameter Nested Logit model (**bottom**). The correct response (4) is increasingly probable as $\theta_{j}$ increases. However, the response category 3—which is the only distractor response where the blue and the yellow squares are (correctly) not adjacent—would be more probably selected by individuals with low abilities ($\theta_{j}\approx -2.7$), while the category 1 would be more probably selected by individuals with even lower abilities ($\theta_{j}<-3$)—thus showing that the choice of distractor may be informative of $\theta_{j}$.

To illustrate NLM, we present in Figure 1 the item-category characteristic curves for an item of the test studied in this very paper.

### 1.3. The Aim of This Study

Although originally, Nominal Response Models were considered as a way to recover information from multiple-choice tests, recent research suggests that, in the case of matrix or series-type GMA tests, NLM may better fit the norminal-level data than the NRM and provide significant reliability gains in comparison with binary logistic models. In particular, Myszkowski and Storme [8] have shown that, on the last series of the Standard Progressive Matrices [18], (1) using NLM provided a better fit than Nominal Response Models to the nominal level data, and (2) NLMs allowed significant reliability gains when estimating ability.

Yet, however promising, this result has only been observed with one GMA test and has only been used on a convenience sample of undergraduates, in a low-stakes situation. This study aims at bridging this gap by replicating this result on another short GMA test, with higher stakes, and in a context that would be particularly interested in these reliability gains: Online recruitment.

The conditions under which job applicants take GMA tests are indeed very different from the conditions in which research participants take similar tests in the lab as part of a typical research study. For example, in a recruitment context, the stakes are higher in comparison with taking the test in a lab. Previous research on the effect of pressure on cognitive processes when taking intelligence tests has shown that when under pressure, working memory is busy processing intrusive thoughts which can have in turn a negative impact on performance [19,20]. It is possible that this phenomenon also affects the way distractors are processed and lead to different processing of response options. When under pressure, test takers who fail at identifying the rule that structures the progression of the series might experience high levels of stress and fail at comparing efficiently distractors to identify the best of the incorrect response options. As a consequence, in the context of the online assessment of job applicants, the choice of distractors might carry little information about the ability of test takers. If this is the case, NLMs should not lead in this context to gains in empirical reliability compared with binary models.

Furthermore, the fact that job applicants usually take online tests in their own time leads to less standardized testing contexts. Compared with the relatively controlled and quiet conditions of a lab, there might be more attentional perturbations in the environment, which might induce a shallower processing of the wrong response options. Consequently, it is possible that in the context of e-assessment, the choice of distractors is not so much reflective of the ability of the test taker, which could hinder the potential gains from NLMs.

The aim of the present study is to test whether the findings of Myszkowski and Storme [8]—obtained in a low psychological pressure and controlled context—can be replicated and generalized to an assessment situation characterized by more psychological pressure and less standardization, as well as a different test.

## 2. Method

### 2.1. Participants and Procedure

The sample consisted of 2949 French job applicants (2084 Men, 865 Women, $M_{age}$ = 36.88, $SD_{age}$ = 8.66) who responded to a logical series test that aims at measuring GMA online. The examinees responded using an e-assessment application presented in their web browser. As it is common in e-assessment, it can be expected that the standardization regarding when and where the test was taken was relatively low as job applicants were free to take the test at the time and at the place that was the most convenient for them. Of the participants, 40.96% had a master (or higher) degree, 23.64% of participants had a bachelor degree, the remaining applicants had less than a bachelor degree.

### 2.2. Instrument

The test under investigation—the GF20—comprises 20 incomplete logical series presented each with six response options to complete the missing part, including one correct answer that can be deducted from the application of logical rules. Each logical series consists of a 4 by 1 matrix with colored cells moving progressively on a grid according to simple geometric rules—such as translations and rotations. The 20 items that are comprised in the final test were designed and pre-tested to discriminate different levels of ability. An item example is provided in Figure 1. Except for instructions participants to complete the series, the test only included non-verbal and non-numerical content. No time limit was provided to applicants to take the test. It took them on average 21.30 min to complete the 20 items (SD = 9.78). Items were presented one by one. Participants were instructed to provide an response to each item before they could move on to the next item, and were not able to go back.

The CTT-based reliability estimates—computed using the R package “semtools” [21] from a unidimensional model fit with the package “lavaan” [22]—of the GF20 were satisfactory, as Cronbach’s α was 0.831, Raykov’s ω congeneric reliability was 0.834 and McDonald’s $\omega_{h}$ reliability was 0.822.

### 2.3. Binary IRT Modeling

#### 2.3.1. Model Estimation

All binary IRT models—the 1-Parameter Logistic (1PL), 2-Parameter Logistic (2PL), 3-Parameter Logistic with free lower asymptote (3PL), and 4-parameter logistic (4PL) models—were estimated using an Expectation-Maximization (EM) algorithm with the R package “mirt” [23]. All models successfully converged. Nevertheless, the information matrix of the 4PL model could not be inverted in order to compute the parameter standard errors—decreasing the convergence tolerance and changing the estimation method did not solve this issue—which may be a sign that the estimates were unstable. Item characteristic curve plots, which, for binary models, present the expected probability of a correct response as a function of the latent ability $\theta_{j}$ were plotted using the package for R “jrt” [24]. To keep the paper concise, only models with appropriate fit were plotted.

#### 2.3.2. Model Fit

The fit of the models were then compared on Likelihood Ratio Tests (LRT) the model’s corrected Akaike Information Criterion (AICc). For the former, *p* values below 0.05 were used to indicate a significantly improved fit from using the more complex (least constrained) model as opposed to the least complex (most constrained) model. For the latter, a smaller AICc indicates a better (more parsimonious) model fit.

In addition, absolute model fit indices were obtained by using limited information Goodness-of-Fit statistics [25] as implemented in “mirt.” As usual—although more frequently seen in Structural Equation Modeling—and similar to the original study of Myszkowski and Storme [8], we used as absolute model fit indices the Comparative Fit Index (CFI) and Tucker-Lewis Index (TLI), with thresholds of 0.95, along with the Standardized Root Mean Square Residual (SRMR) with a threshold of 0.08, and the Root Mean Square Error of Approximation (RMSEA) with a threshold of 0.06.

#### 2.3.3. Reliability

Since the aim of this paper is to extend and replicate the finding that NLM provides an increase in measurement accuracy in logical GMA tests—as found with the Raven’s progressive matrices [8]—quantifying measurement accuracy is key. Measurement accuracy is represented by several statistics in IRT, especially information, standard error of measurement and reliability, which are mathematical transformations of one another. Because reliability is a familiar metric for most researchers—in both CTT and IRT—is conveniently bounded by 0 and 1, and is the metric chosen in the article that this study attempts to replicate, it was chosen in this study. However, it should be noted that the conclusions reached about reliability here are also extendable to information and standard errors.

Similar to the original study, reliability functions were plotted for the 2PL, 3PL and 4PL models, overlayed with their Nested Logit counterparts. In addition, and also similar to the original study, marginal estimates of empirical reliability were computed [26]. The estimate of empirical reliability reported corresponds to the reliability of the $\theta_{j}$ scores, averaged across all cases *j*.

### 2.4. Nominal and Nested Logit IRT Models

#### 2.4.1. Model Estimation

The models for nominal data—the Nominal Response (NR), the 2-Parameter Nested Logit (2PNL), the 3-Parameter Nested Logit (3PNL) an the 4-Parameter Nested Logit (4PNL) models—were estimated using the package “mirt” [23] using an EM algorithm. All models converged successfully. However, as with the binary models, the information matrix of the 4PNL model could not be inverted in order to compute the parameter standard errors, which may be a sign that the estimates were unstable. As for the binary models, item category curve plots, which present the expected probability of selecting a category as a function of the latent ability $\theta_{j}$ were computed using “jrt” [24]. Again, to keep the paper concise, only models with appropriate fit were reported.

#### 2.4.2. Model Fit

Similar to the binary models, Likelihood Ratio Tests were used to compare the different nominal models. However, only the 2PNL, 3PNL and 4PNL models are nested with one another, and thus only they allow the use of Likelihood Ratio Tests when comparing them. The AICcs of all models were computed, and the AICc was used to compare the Nominal Response model with the other models.

Polytomous models are largely more heavily parametrized than binary models, which, in some cases, prevents to compute limited information Goodness-of-Fit statistics, such as in Myszkowski and Storme [8]—thereby limiting model fit estimations. However, in this case, because of the larger sample size than in Myszkowski and Storme [8], we were able to compute them, and used the same indices and thresholds earlier discussed for the binary models.

#### 2.4.3. Reliability

Similar to the binary models, we also computed the reliability functions of the NLMs, which were plotted as an overlay of the reliability functions of their respective binary counterparts (i.e., 2PL and 2PNL, 3PL and 3PLN, 4PL and 4PLN)—thereby facilitating visual comparisons. We also computed the empirical reliability of each model averaged across cases as an estimate of marginal reliability.

As one of the aims of this study is to examine potential gains in reliability from using NLMs as opposed to their binary counterparts, we computed the reliability gain $\Delta r_{xx^{\prime }}$ between models by computing their difference. Similar to the original study and other previous studies [8,15], we used bootstrapping to obtain a Wald’s *z* test (based on the bootstrapped standard error) and 95% Confidence Intervals for the reliability gains.

## 3. Results

### 3.1. Binary IRT Models

The model fit indices of all binary models are reported in Table 1. The 2PL, 3PL and 4PL models all had satisfactory fit, with the 4PL model providing the best fit. The 4PL model fitted significantly better than the 3PL model ($\Delta \chi^{2}=167.405$, Δdf=20, p<0.001), which fitted significantly better than the 2PL model ($\Delta \chi^{2}=519.018$, Δdf=20, p<0.001), which fitted significantly better than the 1PL model ($\Delta \chi^{2}=1100.652$, Δdf=19, p<0.001).

**Figure 2:**
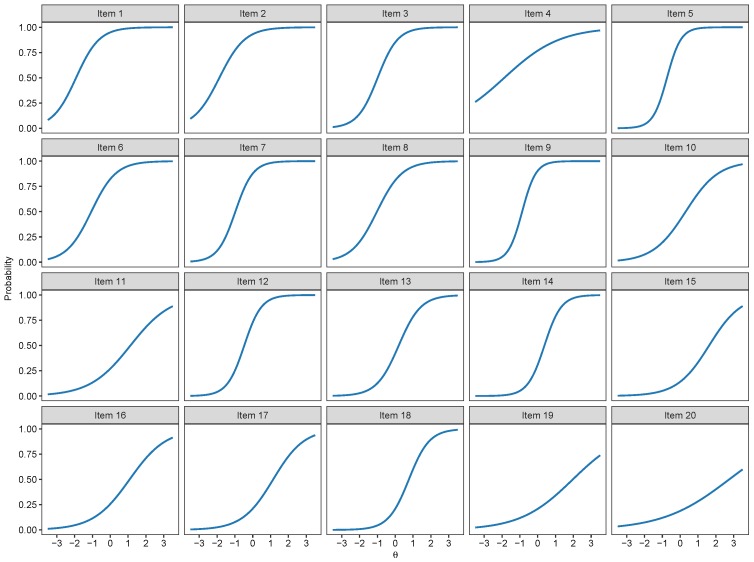
Item characteristic curve plots of the 2-Parameter Logistic Model.

**Figure 3:**
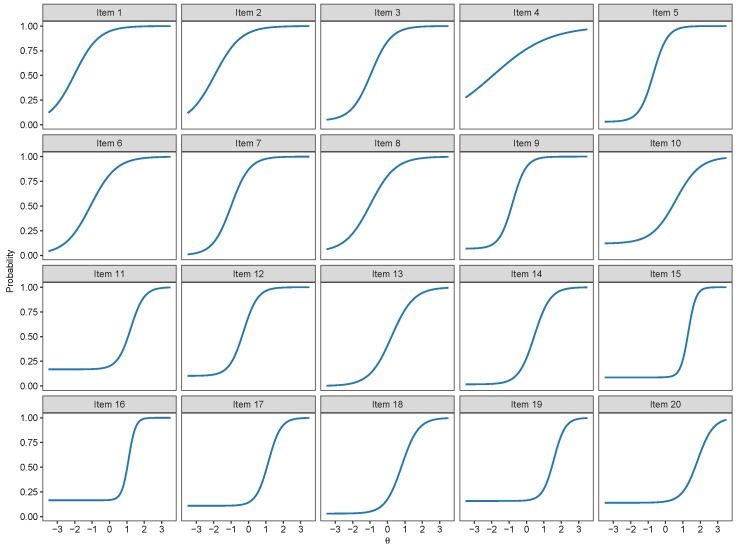
Item characteristic curve plots of the 3-Parameter Logistic Model.

**Figure 4:**
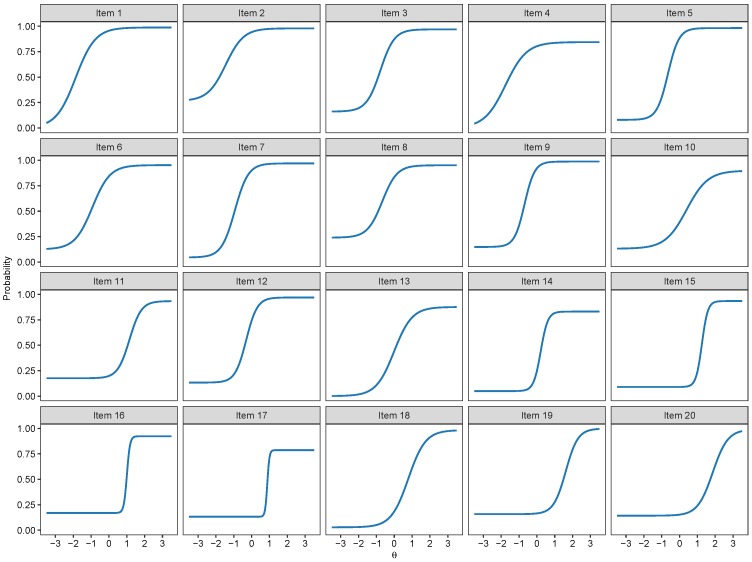
Item characteristic curve plots of the 4-Parameter Logistic Model.

As they were the best two fitting models and provided very similar absolute fit indices, we present the item characteristic curves of both the 2PL, 3PL and 4PL models respectively in Figure 2, Figure 3 and Figure 4. Their similarity and the relatively high low asymptotes for the 4PL model—for the 3PL, they are fixed to 1—are in line with the fact that the two models provided similar fit.

The parameter estimates (along with standard errors for the 2PL and 3PL) of the 2PL, 3PL, and 4PL models are presented respectively in Table 2.

The marginal estimates of empirical reliability for all the binary models were satisfactory and close to the CTT-based estimates earlier reported, as they were 0.833 for the 1PL model, 0.849 for the 2PL model, 0.868 for the 3PL model and 0.873 for the 4PL model.

### 3.2. Nominal Models

The model fit indices of all nominal models are reported in Table 3. Although the Nominal Response model provided a borderline acceptable fit, it was, as hypothesized, outperformed by all the NLMs, which all presented satisfactory fit. The 4PNL model fitted significantly better than the 3PNL model ($\Delta \chi^{2}=82.624$, Δdf=20, p<0.001), which fitted significantly better than the 2PNL model ($\Delta \chi^{2}=541.102$, Δdf=20, p<0.001).

**Figure 5:**
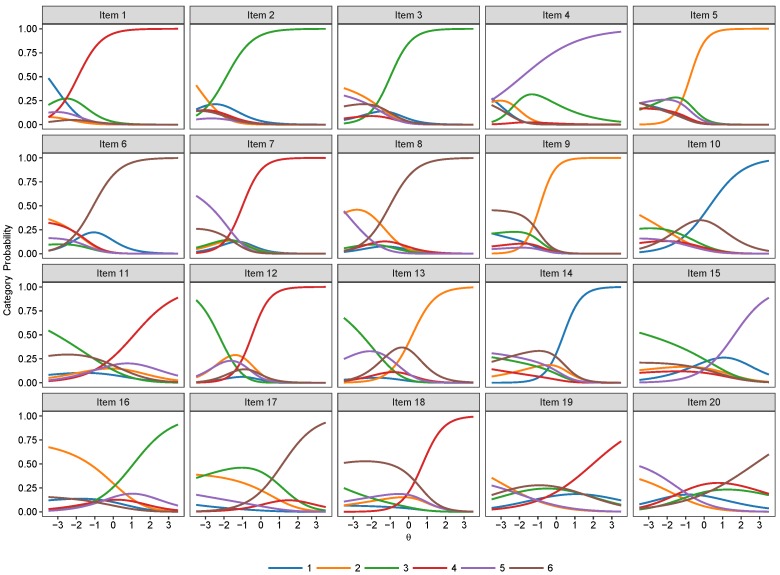
Item category curve plots of the 2-Parameter Nested Logit (2PNL) model.

**Figure 6:**
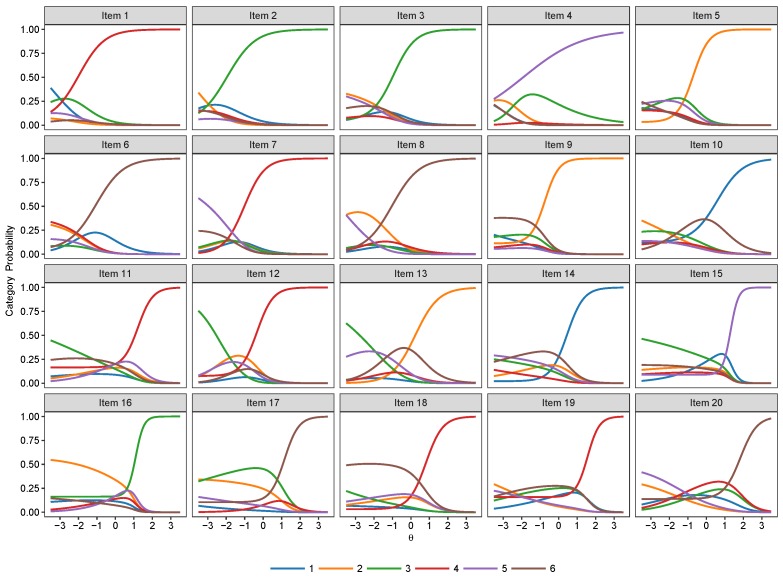
Item category curve plots of the 3-Parameter Nested Logit (3PNL) model.

**Figure 7:**
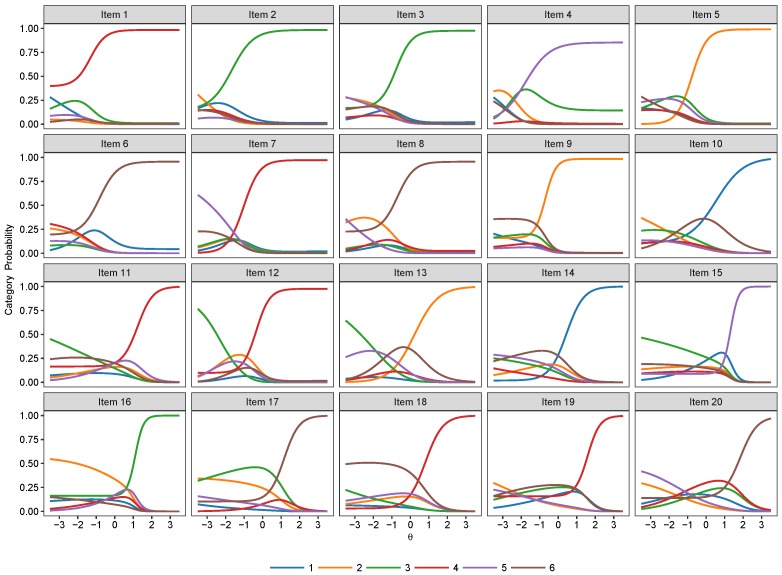
Item category curve plots of the 4-Parameter Nested Logit (4PNL) model.

The item category curve plots of the 2PNL, 3PNL and the 4PNL are respectively presented in Figure 5, Figure 6 and Figure 7. Their model estimates as well as standard errors are presented respectively in Table 4, Table 5 and Table 6.

The marginal estimates of empirical reliability for all the nominal models were satisfactory, as they were 0.857 for the Nominal Response model, 0.867 for the 2PNL model, 0.887 for the 3PNL model and 0.888 for the 4PNL model.

As hypothesized, preferring NLMs instead of binary logistic models resulted in significant reliability gains. The average reliability gains amounted to 0.018 (Bootstrapped 95% CI =[0.017, 0.021], Bootstrapped *z* = 17.765, p<0.001) for the 2PL vs. 2PNL models, 0.019 (Bootstrapped 95% CI =[0.018, 0.023], Bootstrapped *z* = 15.265, p<0.001) for the 3PL vs. 3PNL models, and 0.015 (Bootstrapped 95% CI =[0.011, 0.020], Bootstrapped *z* = 6.669, p<0.001) for the 4PL vs. 4PNL models.

**Figure 8:**
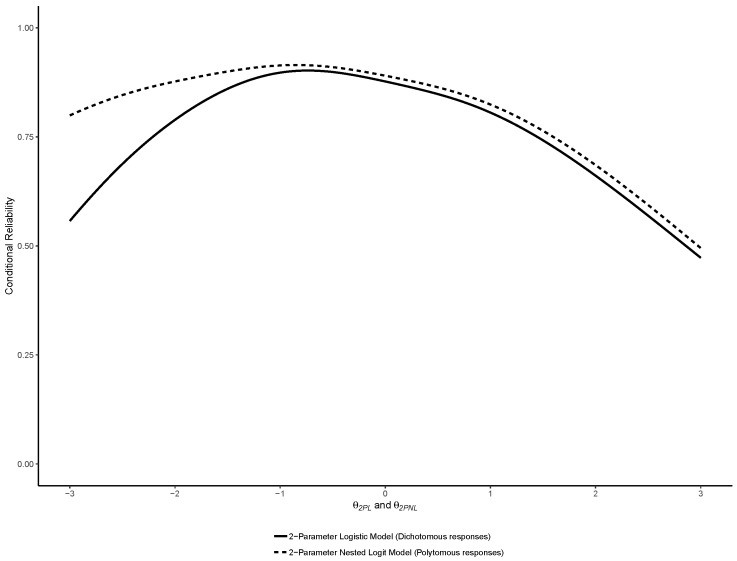
Comparison of the reliability functions of the 2-Parameter Logistic (2PL) and Nested Logit (2PNL) models.

**Figure 9:**
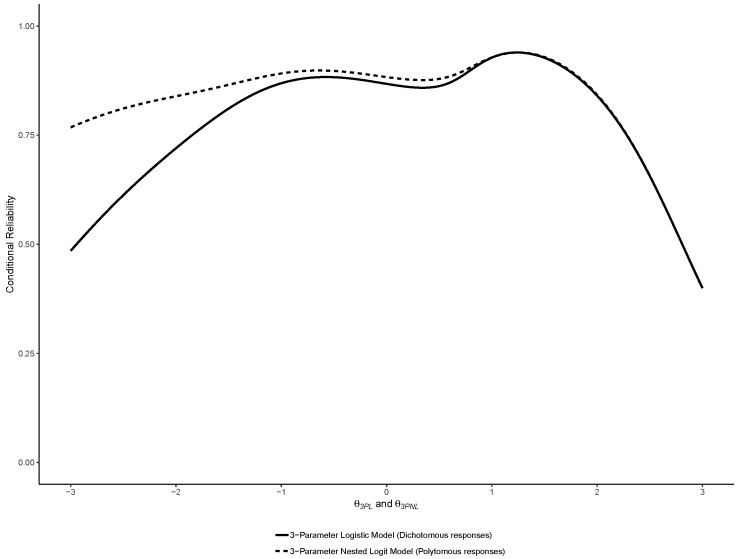
Comparison of the reliability functions of the 3-Parameter Logistic (3PL) and Nested Logit (3PNL) models.

**Figure 10:**
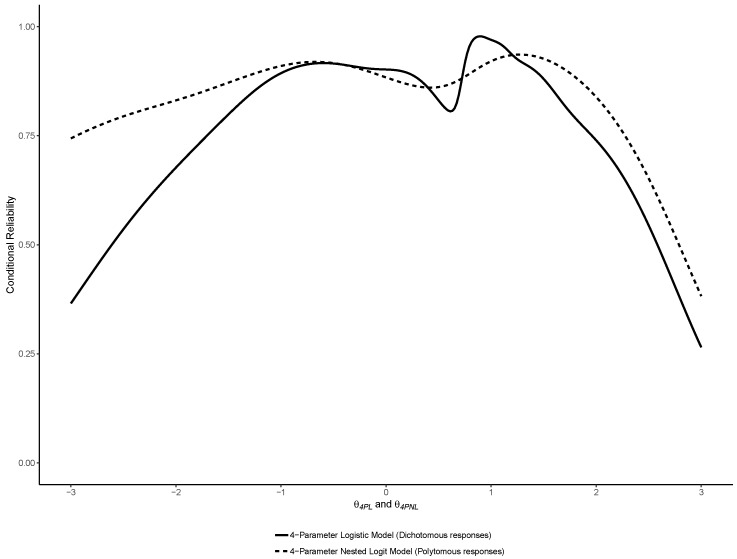
Comparison of the reliability functions of the 4-Parameter Logistic (4PL) and Nested Logit (4PNL) models.

The reliability functions of the 2PL, 3PL and 4PL are reported with their Nested Logit counterparts in respectively Figure 8, Figure 9 and Figure 10. As noted by a reviewer, between a binary model and its nested counterpart, $\theta_{j}$ is not perfectly invariant, and thus the reliability functions may cross, such as in Figure 4. This was also previously observed in the comparison between binary and nominal response models [7].

As expected, they show that using NLM provided increments in reliability especially in the lower range of abilities.

## 4. Discussion

The aim of the present research was to extend the previous findings of Myszkowski and Storme [8] to different testing modalities, online assessment—a different context with higher stakes—and personnel selection, on a larger sample and with a different logical reasoning test.

We found that 4-parameter models—both binary and nested logit—were likely unstable (as their information matrix could not be inverted) but they seemed to outperform their 1PL, 2PL, and 3PL counterparts. Being that the 2-parameter and 3-parameter models did not present this issue while still presenting excellent fit, the results suggest that choosing them may be a more parsimonious but still well fitting approach to this test. In fact, the 2PL and 2PNL fitting respectively almost as well as the 3PL and 3PNL, they may be a more optimal modeling strategy for this test.

We also found that, as hypothesized, Nested Logit Models (NLM) both outperformed the Nominal Response Model [7], providing significant reliability gains compared with their binary counterparts. In addition, the absolute fit of the NLMs—which was not computable in Myszkowski and Storme [8] due to the lower sample size—could be computed here and was found satisfactory, especially regarding the models including a guessing parameter (3PNL and 4PNL).

These findings overall suggest that NLMs [9] are a better modeling alternative than binary logistic models and than the Nominal Response Model [7] for logical reasoning multiple-choice tests, such as incomplete matrix or series tests, in online personnel selection settings.

### 4.1. Theoretical and Practical Implications

From a theoretical viewpoint, the present study can be seen as a conceptual replication and extension of Myszkowski and Storme [8]’s study on Raven’s progressive matrices. Replicating findings is an important endeavor in scientific research. This is especially true in the field of psychology, which is regularly criticized for its lack of consideration for replicating empirical findings [27]. Recently, Hüffmeier et al. [10] have designed a theoretical framework to conceptualize the replication process in psychology and have proposed a typology of replication studies. Rather than considering replication as a process separate from the initial research process, they conceptualize replication as the very research process by which fundamental findings are generalized to situations that are increasingly close to real life conditions.

When a result has been shown at a fundamental level, it may be interesting to replicate it to see if it is not due to chance. In this case, exact or close replications will be used [10]. To be able to further generalize the findings of a fundamental study, it is important to be able to perform conceptual replications in the laboratory or in the field. In conceptual replications, comparability to the original study is limited to the aspects that are considered theoretically relevant [28,29]. Among the conceptual replications are field studies. The aim of such studies is to investigate whether laboratory findings also hold under field conditions, and to rule out the possibility that a laboratory finding is a laboratory artifact or too weak to be relevant in contexts that are less tightly controlled [10]. In the framework described by Hüffmeier et al. [10], our study can be defined as a conceptual replication in the field of the study conducted by Myszkowski and Storme [8]. Our findings suggest that the characteristics of the e-assessment context do not fundamentally affect the way distractors are selected by test takers. Previous basic research on recovering distractor information is therefore relevant in an e-assessment context.

From a practical viewpoint, our findings suggest that one way to improve the accuracy of e-assessment in the context of recruitment is to recover distractor information. Web applications that use tests with distractors should try to implement NLM to get more reliable estimates of the general mental ability of job applicants. To this day, there are few software implementations of NLM. A recommendation to designers of IRT platforms would be to add NLM to their offer. For e-assessment platforms, a relatively inexpensive alternative to commercial IRT software could be to use the “mirt” [23] R library on the server side to estimate the ability of test takers using the built-in NLM function. One of the challenges of this option is that R can be a programming language that is relatively consuming in terms of computing resources and time, although $\theta_{j}$ estimations in “mirt” are relatively fast once the parameters of the model are stored in memory. More optimizations that will facilitate the implementation of NLM in e-assessment might come in the future.

In line with the findings of Myszkowski and Storme [8], the observed gain in reliability was especially visible at relatively low levels of ability. This is not surprising as NLM recover information from wrong response options. Recruiters are usually interested in applicants with high levels of intelligence, but this is not always the case. For example, it is possible that due to high competition on the job market, a recruiter is unable to attract the best applicants, and has to select among applicants with relatively lower levels of ability. In such situations, the use of NLM could be highly valuable as it allows forming a more accurate impression of applicants on the low end of the trait, and selecting the best.

As a reviewer pointed out, the standard errors of item parameter estimates of the Nested Logit Models were overall smaller than their binary counterparts—this of course only concerns parameters that are common between models (difficulty, discrimination and, for the 3PL and 3PNL, guessing). This result may seem counterintuitive, because, in general, for a given dataset, item parameter standard errors tend to increase as model complexity increases, and the Nested Logit Models are substantially more parametrized than the binary models. However, it should be noted that the Nested Logit Models are not only more complex, but they also use, to some extent, a different dataset, in that they use more information from the base dataset. Indeed, they use the complete information from the nominal level, while binary models use only the information at the binary level. Although we have showed that, like in Myszkowski and Storme [8], Nested Logit Models resulted in gains in reliability (and thus lower standard errors) for the person estimates, the present results also suggest that the difficulty, disscrimination and guessing parameters of the Nested Logit Models are estimated with more accuracy—because they use more information—than the respective item parameters of their binary counterparts. This result calls for replication in other datasets, contexts and types of tests.

Throughout the paper, we have mostly emphasized the benefits of using NLM to improve the accuracy of ability estimates. However, NLM has other potentially interesting applications beyond improving scoring. For example, Suh and Bolt [30] have described a method relying on NLM to evaluate how distractors might contribute to Differential Item Functioning (DIF) [30]. It is indeed possible that distractors function differently across groups, leading to Differential Distractor Functioning (DDF). DDF can lead in turn to DIF, which is a major problem when using the same test on different groups. Multigroup NLM could help test designers to improve the diagnosis of the causes of DIF, and thus to improve their tests. Bolt et al. [31] have suggested another interesting application of NLM, which is to use NLM as a way to determine whether the ability distinguished by distractors is the same as the ability underlying the choice of the correct response. Here again, the use of NLM could help test designers to select items that best reflect the underlying ability.

### 4.2. Limitations and Future Research

Our study has several limitations which should stimulate and guide further research on the topic. A first limitation is related to the sample that was used in the study. The sample comes from a single e-assessment platform and it is therefore difficult to know whether the findings would generalize to other platforms. It is possible for example that characteristics of the design of Web applications affect the way distractors are processed by test takers. Previous research has shown that the experience of users greatly affect the cognitive processes they mobilize when using a Web application [32]. Applied to our question, it is possible that a bad Web design reduces the motivation of test takers to process distractors when they fail at identifying the rule governing the logical progression of the series. Further research is needed to test the generalizability of the findings to other platforms, but also to other types of GMA tasks.

Antother limitation is related to our sample size. NLM have more parameters than the models to which they were compared in the current study. Although our sample is larger than the one used in the original study that we conceptually replicated [8], it is still unclear whether our sample size is large enough to get reliable parameter estimates. Further research using Monte-Carlo simulations is needed on the influence of sample size on parameter estimation in NLM, and to provide clear guidelines regarding the necessary sample size.

In addition, it should be noted that the fact that NLM provided a better fit, like in Myszkowski and Storme [8]’s study, does not necessarily imply that the cognitive processes engaged in responding similar tests are necessarily only the 2-step sequence that the NLM are based on—attempting to solve the task by looking at the stimulus only and then, if the correct answer is not found, examining the distractors. Indeed, it remains very possible that the actual responding process is less clear and closer to a back-and-forth between a stimulus-based strategy and a response option comparison-based strategy. Further, it has been noted that NLM may be further improved by including the possibility that the guessing strategy (level 2) results in the choice of the correct response. In other words, choosing the correct response could then be the result or either strategy. Future research might consider this interesting possibility when such models are available in traditional IRT software.

Another limitation of this study is that it was limited in the breadth of nominal models tested by their availibility in “mirt.” Although this package provides a large number of popular models, we were not able to fit some alternatives models, notably Thissen and Steinberg [33]’s Multiple Choice Model (MCM), which essentially adds to the Nominal Response model a latent state category for examinees that corresponds to an examinee not knowing—and thus guessing—what the correct response is. Although the Nominal Response model was here outperformed by the Nested Logit models, it may be that alternative models like the MCM are better alternatives.

Another important limitation of our study is that we did not test whether the improvement in reliability translates into an improvement in predictive validity. This is because our study did not include a measure of job performance. The ability of an assessment tool to predict future job performance is crucial in the context of recruitment. Improvements in measurement reliability can lead to improvements in predictive validity, as reliability is a prerequisite for validity [34].

Whether recovering distractor information actually improves predictive validity in the context of e-assessment remains to be investigated. The answer to this question could represent an important contribution to the literature. It has indeed been shown that in situations in which test takers are under pressure, for example when stakes are high, the predictive validity of GMA tests tends to decrease [35]. Duckworth et al. [35] argued that GMA tests predict various indicators of success in life because when they are used in low stakes contexts, they essentially measure the motivation of test takers. According to Duckworth et al. [35], it is because GMA tests taken in the lab measure motivation that they are found to be positively associated with a broad range of indicators of life success. Although there is empirical evidence supporting Duckworth et al. [35]’s argument, one can wonder whether using a more precise strategy to score GMA tests could not ultimately reveal that there is a relation between GMA and various indicators of achievement. Testing the predictive validity of GMA tests scored with NLM could therefore have important implications regarding the knowledge of the true relationship between GMA and achievement in general.

## Figures and Tables

**Table 1:** Model fit of the binary models.

Model	$\chi^{2}$	df	*p*	CFI	TLI	RMSEA	AICc
1-Parameter Logistic	2462.597	189	<0.001	0.913	0.913	0.064	58,244.74
2-Parameter Logistic	1069.812	170	<0.001	0.966	0.962	0.042	57,182.90
3-Parameter Logistic	251.3807	150	<0.001	0.996	0.995	0.015	56,705.29
4-Parameter Logistic	196.2342	130	<0.001	0.997	0.996	0.013	56,579.87

**Table 2:** Item parameters of binary logistic models.

Item	1PL Model	2PL Model		3PL Model			4PL Model			
	$\beta_{i}$	$\alpha_{i}$	$\beta_{i}$	$\alpha_{i}$	$\beta_{i}$	logit($\gamma_{i}$)	$\alpha_{i}$	$\beta_{i}$	logit($\gamma_{i}$)	logit($\delta_{i}$)
**Item 1**										
Estimate	2.783	1.527	2.930	1.417	2.855	−4.089	1.821	3.391	0.002	0.988
Standard error	0.072	0.105	0.113	0.104	0.157	6.671				
**Item 2**										
Estimate	2.569	1.391	2.605	1.326	2.575	−5.002	1.999	2.898	0.266	0.979
Standard error	0.068	0.094	0.096	0.087	0.104	6.298				
**Item 3**										
Estimate	1.513	1.767	1.740	1.735	1.633	−3.170	2.558	1.988	0.161	0.969
Standard error	0.055	0.093	0.078	0.155	0.136	2.191				
**Item 4**										
Estimate	1.454	0.640	1.198	0.619	1.185	−5.598	1.697	2.979	0.002	0.844
Standard error	0.055	0.054	0.048	0.051	0.057	6.083				
**Item 5**										
Estimate	1.291	2.543	1.878	2.462	1.719	−3.465	3.222	2.097	0.080	0.982
Standard error	0.053	0.132	0.099	0.179	0.102	1.223				
**Item 6**										
Estimate	1.475	1.442	1.535	1.362	1.477	−4.939	2.028	1.880	0.125	0.952
Standard error	0.055	0.078	0.067	0.083	0.089	6.479				
**Item 7**										
Estimate	1.588	2.015	1.966	1.891	1.884	−6.457	2.667	2.501	0.045	0.969
Standard error	0.056	0.106	0.089	0.097	0.084	6.179				
**Item 8**										
Estimate	1.404	1.412	1.448	1.389	1.365	−3.355	2.415	1.622	0.240	0.951
Standard error	0.054	0.077	0.064	0.141	0.178	3.531				
**Item 9**										
Estimate	1.542	2.575	2.245	2.593	2.061	−2.619	3.540	2.450	0.148	0.987
Standard error	0.055	0.138	0.111	0.216	0.118	0.751				
**Item 10**										
Estimate	−0.372	1.085	−0.335	1.438	−0.852	−2.002	1.683	−0.669	0.131	0.898
Standard error	0.050	0.059	0.046	0.149	0.172	0.285				
**Item 11**										
Estimate	−1.137	0.878	−0.991	2.603	−3.188	−1.597	3.013	−3.462	0.176	0.934
Standard error	0.052	0.055	0.048	0.327	0.400	0.093				
**Item 12**										
Estimate	0.762	2.078	0.991	2.440	0.697	−2.171	3.235	0.976	0.133	0.969
Standard error	0.051	0.101	0.070	0.177	0.099	0.276				
**Item 13**										
Estimate	−0.313	1.612	−0.316	1.577	−0.368	−7.978	1.988	0.021	0.001	0.876
Standard error	0.049	0.079	0.054	0.076	0.055	6.064				
**Item 14**										
Estimate	−0.662	2.121	−0.802	2.191	−0.992	−4.072	5.037	−1.078	0.049	0.831
Standard error	0.050	0.105	0.067	0.146	0.106	0.616				
**Item 15**										
Estimate	−1.926	1.113	−1.807	4.520	−5.921	−2.352	6.060	−7.593	0.090	0.934
Standard error	0.059	0.068	0.066	0.608	0.762	0.090				
**Item 16**										
Estimate	−1.186	0.981	−1.064	5.056	−5.569	−1.622	11.675	−11.750	0.169	0.923
Standard error	0.053	0.058	0.051	0.754	0.841	0.071				
**Item 17**										
Estimate	−1.399	1.153	−1.321	2.815	−3.228	−2.099	16.917	−14.883	0.132	0.787
Standard error	0.054	0.065	0.057	0.311	0.353	0.111				
**Item 18**										
Estimate	−1.192	1.736	−1.330	2.104	−1.729	−3.465	2.079	−1.636	0.028	0.983
Standard error	0.053	0.089	0.068	0.156	0.143	0.343				
**Item 19**										
Estimate	−1.603	0.678	−1.335	3.085	−4.858	−1.673	2.931	−4.739	0.158	0.998
Standard error	0.056	0.054	0.050	0.520	0.759	0.077				
**Item 20**										
Estimate	−1.808	0.532	−1.463	2.248	−4.156	−1.817	2.369	−4.404	0.143	0.990
Standard error	0.058	0.053	0.050	0.372	0.580	0.089				

**Table 3:** Model fit of the nominal and nested logit models.

Model	$\chi^{2}$	df	*p*	CFI	TLI	RMSEA	AICc
Nominal Response	178.0345	90	<0.001	0.972	0.941	0.018	134,347.1
2-Parameter Nested Logit	177.3853	90	<0.001	0.978	0.958	0.018	133,725.8
3-Parameter Nested Logit	126.1003	70	<0.001	0.986	0.965	0.016	133,231.1
4-Parameter Nested Logit	104.8853	50	<0.001	0.986	0.952	0.019	133,195.5

**Table 4:** Item parameters of the 2PNL model.

Item	Correct Response		Distractors							
	$\alpha_{i}$	$\beta_{i}$	$\lambda_{i,1}$	$\lambda_{i,2}$	$\lambda_{i,3}$	$\lambda_{i,4}$	$\delta_{i,1}$	$\delta_{i,2}$	$\delta_{i,3}$	$\delta_{i,4}$
**Item 1**										
Estimate	1.549	2.954	0.426	1.067	0.744	1.327	−0.312	2.878	1.248	1.771
Standard error	0.103	0.113	0.535	0.341	0.383	0.382	0.805	0.523	0.579	0.554
**Item 2**										
Estimate	1.397	2.614	−1.189	−0.297	−0.123	−0.442	−3.219	−1.154	−1.509	−1.669
Standard error	0.091	0.096	0.357	0.194	0.229	0.231	0.548	0.235	0.266	0.292
**Item 3**										
Estimate	1.747	1.736	−0.915	−0.392	−0.914	−0.559	−1.147	−1.094	−1.369	−0.593
Standard error	0.089	0.077	0.183	0.193	0.195	0.163	0.205	0.197	0.222	0.167
**Item 4**										
Estimate	0.646	1.200	0.828	2.623	2.413	0.132	2.746	6.883	4.011	0.168
Standard error	0.053	0.048	0.650	0.646	0.671	0.821	1.150	1.122	1.136	1.476
**Item 5**										
Estimate	2.417	1.819	0.648	0.180	0.386	−0.077	1.893	0.351	1.343	−0.259
Standard error	0.119	0.093	0.215	0.255	0.221	0.279	0.260	0.313	0.270	0.355
**Item 6**										
Estimate	1.412	1.524	−1.426	−1.127	−1.334	−1.266	−2.516	−2.816	−2.308	−2.755
Standard error	0.075	0.066	0.205	0.244	0.194	0.231	0.245	0.283	0.226	0.274
**Item 7**										
Estimate	1.945	1.933	−0.373	−0.506	−1.447	−1.262	−0.445	−0.617	−1.647	−1.845
Standard error	0.098	0.085	0.171	0.179	0.218	0.237	0.171	0.184	0.267	0.291
**Item 8**										
Estimate	1.425	1.457	−0.868	−0.485	−0.010	−1.611	0.098	−0.573	0.507	−2.450
Standard error	0.075	0.064	0.163	0.194	0.153	0.288	0.161	0.189	0.136	0.384
**Item 9**										
Estimate	2.435	2.170	0.354	0.517	0.485	0.233	1.225	0.780	0.208	1.577
Standard error	0.123	0.103	0.244	0.270	0.307	0.230	0.303	0.325	0.365	0.291
**Item 10**										
Estimate	1.090	−0.336	0.327	0.472	0.248	1.177	0.701	0.359	−0.068	2.060
Standard error	0.058	0.046	0.131	0.144	0.155	0.123	0.137	0.145	0.161	0.122
**Item 11**										
Estimate	0.875	−0.991	0.318	−0.397	0.567	−0.116	0.613	0.501	0.834	0.817
Standard error	0.055	0.048	0.109	0.107	0.107	0.103	0.088	0.093	0.085	0.086
**Item 12**										
Estimate	2.087	0.992	−0.374	−1.895	−0.589	0.182	1.101	−1.492	0.542	1.008
Standard error	0.098	0.069	0.195	0.264	0.206	0.202	0.168	0.306	0.185	0.167
**Item 13**										
Estimate	1.626	−0.321	−0.718	0.558	−0.139	0.922	0.555	1.467	1.580	2.877
Standard error	0.078	0.055	0.216	0.211	0.201	0.196	0.226	0.197	0.197	0.185
**Item 14**										
Estimate	2.096	−0.804	−0.577	−0.696	−0.539	−0.221	−0.613	−1.659	−0.334	0.435
Standard error	0.102	0.067	0.114	0.158	0.107	0.092	0.097	0.147	0.089	0.070
**Item 15**										
Estimate	1.104	−1.803	−0.520	−0.781	−0.564	−0.680	−0.343	0.130	−0.730	−0.432
Standard error	0.067	0.065	0.087	0.078	0.096	0.089	0.067	0.061	0.075	0.070
**Item 16**										
Estimate	0.965	−1.060	−0.187	0.467	0.802	−0.199	1.074	0.209	0.407	−0.445
Standard error	0.057	0.050	0.092	0.113	0.112	0.125	0.076	0.086	0.084	0.106
**Item 17**										
Estimate	1.118	−1.309	0.310	0.512	1.364	0.149	2.761	3.379	1.632	1.423
Standard error	0.064	0.056	0.196	0.193	0.217	0.212	0.189	0.187	0.201	0.204
**Item 18**										
Estimate	1.781	−1.351	0.400	−0.291	0.321	0.097	1.451	0.316	1.619	2.397
Standard error	0.090	0.069	0.156	0.175	0.152	0.144	0.131	0.159	0.129	0.124
**Item 19**										
Estimate	0.675	−1.335	−0.936	−0.235	−0.812	−0.294	−1.112	0.342	−0.935	0.431
Standard error	0.053	0.050	0.110	0.074	0.104	0.073	0.103	0.061	0.094	0.060
**Item 20**										
Estimate	0.533	−1.463	−0.720	0.390	0.318	−0.680	−1.051	0.208	0.541	−0.578
Standard error	0.053	0.050	0.110	0.079	0.074	0.095	0.103	0.064	0.060	0.087

**Table 5:** Item parameters of the 3PNL model.

Item	Correct Response			Distractors							
	$\alpha_{i}$	$\beta_{i}$	logit($\gamma_{i}$)	$\lambda_{i,1}$	$\lambda_{i,2}$	$\lambda_{i,3}$	$\lambda_{i,4}$	$\delta_{i,1}$	$\delta_{i,2}$	$\delta_{i,3}$	$\delta_{i,4}$
**Item 1**											
Estimate	1.443	2.843	−3.065	0.396	0.927	0.668	1.132	−0.323	2.768	1.196	1.623
Standard error	0.125	0.231	4.501	0.474	0.304	0.342	0.343	0.761	0.500	0.552	0.532
**Item 2**											
Estimate	1.319	2.564	−4.277	−1.109	−0.271	−0.132	−0.452	−3.220	−1.141	−1.522	−1.709
Standard error	0.086	0.111	4.167	0.323	0.174	0.207	0.209	0.540	0.226	0.259	0.288
**Item 3**											
Estimate	1.719	1.608	−2.921	−0.824	−0.424	−0.883	−0.496	−1.086	−1.130	−1.378	−0.549
Standard error	0.149	0.131	1.645	0.164	0.176	0.177	0.146	0.194	0.193	0.216	0.158
**Item 4**											
Estimate	0.625	1.181	−4.813	0.684	2.387	2.171	−0.076	2.602	6.763	3.888	−0.230
Standard error	0.051	0.065	4.023	0.588	0.581	0.606	0.769	1.136	1.100	1.115	1.535
**Item 5**											
Estimate	2.340	1.664	−3.412	0.536	0.127	0.300	−0.207	1.795	0.298	1.263	−0.421
Standard error	0.168	0.098	1.215	0.191	0.227	0.197	0.252	0.242	0.293	0.252	0.343
**Item 6**											
Estimate	1.363	1.418	−3.134	−1.265	−0.982	−1.262	−1.159	−2.399	−2.706	−2.295	−2.695
Standard error	0.125	0.158	2.550	0.183	0.217	0.176	0.208	0.230	0.264	0.220	0.263
**Item 7**											
Estimate	1.826	1.854	−6.090	−0.338	−0.431	−1.327	−1.119	−0.425	−0.565	−1.599	−1.751
Standard error	0.090	0.081	4.208	0.155	0.160	0.197	0.213	0.165	0.174	0.259	0.278
**Item 8**											
Estimate	1.378	1.394	−4.012	−0.752	−0.427	−0.002	−1.495	0.168	−0.543	0.512	−2.442
Standard error	0.103	0.123	4.313	0.147	0.176	0.141	0.263	0.154	0.183	0.133	0.381
**Item 9**											
Estimate	2.648	1.969	−2.061	0.408	0.533	0.422	0.297	1.305	0.828	0.176	1.664
Standard error	0.200	0.116	0.370	0.225	0.249	0.285	0.212	0.298	0.320	0.364	0.286
**Item 10**											
Estimate	1.461	−0.870	−1.983	0.317	0.456	0.250	1.137	0.701	0.356	−0.061	2.034
Standard error	0.152	0.172	0.274	0.119	0.132	0.141	0.113	0.133	0.141	0.156	0.118
**Item 11**											
Estimate	2.527	−3.084	−1.619	0.279	−0.374	0.581	−0.113	0.605	0.515	0.824	0.819
Standard error	0.315	0.385	0.096	0.106	0.102	0.106	0.099	0.087	0.092	0.085	0.085
**Item 12**											
Estimate	2.308	0.758	−2.504	−0.344	−1.748	−0.542	0.148	1.120	−1.412	0.573	0.990
Standard error	0.159	0.093	0.359	0.180	0.243	0.191	0.188	0.162	0.296	0.178	0.161
**Item 13**											
Estimate	1.593	−0.374	−7.481	−0.630	0.525	−0.116	0.852	0.615	1.446	1.596	2.837
Standard error	0.075	0.055	3.982	0.194	0.191	0.181	0.177	0.216	0.189	0.189	0.177
**Item 14**											
Estimate	2.249	−1.038	−3.833	−0.510	−0.646	−0.473	−0.183	−0.576	−1.635	−0.297	0.452
Standard error	0.142	0.102	0.434	0.104	0.144	0.098	0.085	0.094	0.143	0.086	0.069
**Item 15**											
Estimate	4.703	−6.146	−2.335	−0.596	−0.800	−0.590	−0.707	−0.344	0.144	−0.721	−0.422
Standard error	0.663	0.831	0.089	0.088	0.079	0.097	0.089	0.067	0.061	0.075	0.070
**Item 16**											
Estimate	4.626	−5.091	−1.638	−0.152	0.446	0.824	−0.214	1.089	0.205	0.404	−0.452
Standard error	0.608	0.675	0.072	0.088	0.112	0.115	0.118	0.075	0.086	0.084	0.105
**Item 17**											
Estimate	2.613	−3.013	−2.142	0.328	0.520	1.452	0.162	2.774	3.387	1.618	1.434
Standard error	0.277	0.313	0.117	0.182	0.180	0.211	0.198	0.188	0.186	0.201	0.202
**Item 18**											
Estimate	2.210	−1.798	−3.415	0.377	−0.242	0.321	0.122	1.444	0.342	1.618	2.407
Standard error	0.159	0.143	0.300	0.144	0.160	0.141	0.133	0.129	0.156	0.127	0.122
**Item 19**											
Estimate	3.167	−4.950	−1.672	−0.901	−0.241	−0.773	−0.300	−1.090	0.344	−0.911	0.434
Standard error	0.523	0.760	0.076	0.104	0.076	0.100	0.074	0.101	0.061	0.092	0.060
**Item 20**											
Estimate	2.233	−4.111	−1.827	−0.659	0.381	0.315	−0.628	−1.018	0.205	0.537	−0.551
Standard error	0.357	0.552	0.089	0.102	0.079	0.073	0.089	0.100	0.064	0.060	0.084

**Table 6:** Item parameters of the 4PNL model.

Item	Correct Response				Distractors							
	$\alpha_{i}$	$\beta_{i}$	logit($\gamma_{i}$)	logit($\delta_{i}$)	$\lambda_{i,1}$	$\lambda_{i,2}$	$\lambda_{i,3}$	$\lambda_{i,4}$	$\delta_{i,1}$	$\delta_{i,2}$	$\delta_{i,3}$	$\delta_{i,4}$
**Item 1**												
Estimate	2.447	3.234	0.395	0.983	0.413	0.955	0.677	1.174	−0.314	2.778	1.189	1.640
**Item 2**												
Estimate	1.733	2.875	0.149	0.983	−1.077	−0.283	−0.142	−0.448	−3.143	−1.150	−1.530	−1.697
**Item 3**												
Estimate	2.364	1.832	0.168	0.975	−0.801	−0.428	−0.904	−0.482	−1.052	−1.132	−1.392	−0.534
**Item 4**												
Estimate	1.517	2.702	0.016	0.852	0.690	2.391	2.175	0.049	2.619	6.790	3.913	0.015
**Item 5**												
Estimate	2.474	1.897	0.001	0.990	0.500	0.121	0.258	−0.309	1.749	0.288	1.212	−0.542
**Item 6**												
Estimate	2.063	1.698	0.192	0.956	−1.277	−1.048	−1.307	−1.155	−2.392	−2.766	−2.329	−2.673
**Item 7**												
Estimate	2.323	2.377	0.002	0.972	−0.336	−0.421	−1.341	−1.087	−0.424	−0.556	−1.613	−1.706
**Item 8**												
Estimate	2.260	1.575	0.225	0.955	−0.765	−0.426	0.005	−1.551	0.166	−0.539	0.515	−2.474
**Item 9**												
Estimate	3.508	2.443	0.159	0.985	0.447	0.560	0.460	0.325	1.343	0.851	0.212	1.691
**Item 10**												
Estimate	1.357	−0.757	0.104	0.999	0.332	0.473	0.278	1.152	0.711	0.368	−0.041	2.048
**Item 11**												
Estimate	2.444	−3.023	0.165	1.000	0.286	−0.378	0.583	−0.110	0.608	0.512	0.829	0.820
**Item 12**												
Estimate	2.766	0.948	0.098	0.976	−0.327	−1.817	−0.519	0.160	1.131	−1.481	0.590	0.997
**Item 13**												
Estimate	1.576	−0.356	0.000	1.000	−0.640	0.555	−0.096	0.890	0.607	1.466	1.611	2.861
**Item 14**												
Estimate	2.176	−0.980	0.019	1.000	−0.510	−0.661	−0.469	−0.177	−0.579	−1.646	−0.297	0.453
**Item 15**												
Estimate	4.743	−6.281	0.088	1.000	−0.585	−0.799	−0.588	−0.704	−0.348	0.136	−0.727	−0.428
**Item 16**												
Estimate	4.613	−5.115	0.162	1.000	−0.155	0.454	0.830	−0.225	1.087	0.210	0.413	−0.458
**Item 17**												
Estimate	2.496	−2.914	0.104	1.000	0.357	0.555	1.501	0.196	2.792	3.409	1.641	1.454
**Item 18**												
Estimate	2.146	−1.745	0.031	1.000	0.359	−0.264	0.303	0.102	1.437	0.332	1.611	2.398
**Item 19**												
Estimate	3.048	−4.844	0.157	0.998	−0.912	−0.245	−0.784	−0.300	−1.096	0.343	−0.917	0.433
**Item 20**												
Estimate	2.217	−4.113	0.139	0.991	−0.666	0.393	0.319	−0.633	−1.023	0.206	0.540	−0.555
